# Tunable Conversion of Ammonia to Hydrazine or Ammonium Nitrite Induced by Acoustic Cavitation Bubbles

**DOI:** 10.1002/cssc.70847

**Published:** 2026-07-05

**Authors:** Damien Denis, Zhangyue Xie, Elodie Fourré, Prince N. Amaniampong, Wen Liu, François Jérôme

**Affiliations:** ^1^ Institut de Chimie des Milieux et Matériaux de Poitiers CNRS, Université de Poitiers Poitiers France; ^2^ School of Chemistry Chemical Engineering and Biotechnology Nanyang Technological University Singapore Singapore

**Keywords:** ammonia, cavitation bubble, hydrazine, nitrite, ultrasound

## Abstract

In this work, we explore the possibility to harness the energy released during the implosion of acoustic cavitation bubbles to activate and selectively convert NH_3_ in water without the assistance of any catalyst. Two primary products, hydrazine and ammonium nitrite, were identified in the liquid phase. Hydrazine was formed as a result of the cleavage of the N─H bond of NH_3_ at the cavitation bubble collapse time, while ammonium nitrite was formed through oxidation of NH_3_ with HO^●^ radicals stemming from the sonolysis of water. By adjusting the gas atmosphere, the generation of HO^●^ radicals can be suppressed or enhanced, thereby enabling selectivity control over the sonochemical conversion pathway of NH_3_ toward either hydrazine or ammonium nitrite. For instance, we show that under H_2_ atmosphere, NH_3_ is predominantly converted to hydrazine, while under oxygen atmosphere ammonium nitrite becomes the major product formed. Finally, we discuss the unexpected beneficial role of salts (mono‐, di‐, and trivalent), which influence the efficiency of this sonochemical activation of NH_3_.

## Graphical abstract

We show here that, depending on the gaseous atmosphere used, energy released at the acoustic cavitation bubble collapse time can be harnessed to activate and selectively convert ammonia into either hydrazine or ammonium nitrite, without assistance of any catalyst, “simply” through the application of ultrasonic waves.

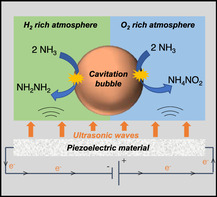

## 1 Introduction

Ammonia (NH_3_), with an annual production of about 160 million tons [1, 2], is the most abundant amine and is nowadays widely used in the chemical industry. It serves as a building block for manufacturing a broad range of products, including fertilizers, surfactants, monomers, solvents, and pharmaceuticals [3, 4]. Driven by the energy transition, NH_3_ is also gaining attention as a promising molecule for hydrogen storage and release [5, 8]. During its transformation, the activation of NH_3_ is often the most critical step, as the N─H bond dissociation energy of NH_3_ is as high as 415 kJ·mol^−1^ [9, 10]. Traditionally, NH_3_ activation and conversion are achieved catalytically over transition metals through mechanisms such as (i) deprotonation [11, 12], (ii) oxidative addition [13, 16], or (iii) hydrogen atom or electron transfer [17, 18].

The urgent need to move away from fossil energy in chemical processes has catalyzed the exploration of alternative technologies [19] (e.g., electrochemistry [20, 24], photochemistry [18, 25, 33], electron beam [34, 37], plasma [38, 46], etc.) for the activation and conversion of NH_3_, with the ultimate goal of achieving low‐carbon processes through electrification or use of solar energy. Among them, the possibility to harness the energy released from the implosion of cavitation bubbles, formed by ultrasonic irradiation of aqueous solution at a high frequency, to induce chemical reactions has gaining more and more interest [47, 48]. In this context, we recently demonstrated that the N─H bond of NH_3_ can undergo cleavage at the proximity of cavitation bubbles generated by high‐frequency ultrasonic irradiation of water, leading to the formation of biradical NH species that recombine to produce hydrazine, a compound of major industrial importance [49, 50]. This work represents the first example of direct NH_3_‐to‐hydrazine conversion, a reaction thermodynamically unfeasible *via* conventional catalysis, highlighting the breakthrough potential of cavitation bubbles in this field. In this work, we also revealed that a 5 wt% NH_3_ concentration in H_2_O is optimal for achieving the highest hydrazine production rate. Here, we go further by analyzing the nitrogen‐containing byproducts generated in both liquid and gas phases, such as nitrite, nitrate, N_2_O, and NO_*x*_, during the sonochemical conversion of NH_3_ to hydrazine. Given the environmental hazards posed by ammonium nitrite and related NO_*x*_ compounds, we explored how the gaseous atmosphere (notably air, O_2_ vs. H_2_) influences the sonochemical conversion of NH_3_. Our key finding, and the most original aspect of this work, is the development of a selective method for converting ammonia into hydrazine, without side formation of harmful ammonium nitrite and NO_*x*_ byproducts. We also investigated here the impact of the water purity, in particular the role of some dissolved salts, on the initial formation rate of hydrazine.

## 2 Results and Discussion

### 2.1 Hydrazine Formation

Following our previous work [49, 50], the sonochemical activation/conversion of NH_3_ (5 wt% in water) was performed at 525 kHz in a SINAPTEC ultrasonic reactor. During ultrasonic irradiation, the reactor temperature was maintained at 45 °C by circulating a cooling liquid (30 °C) through the reactor double jacket. Further details of the ultrasonic reactor can be found in the electro spray ionization (ESI) (Figure S1). Because the present ultrasonic reactor differs in both size and power from the one used in our previous study, we first repeated the sonochemical activation and conversion of NH_3_ using our previously reported optimized conditions (100 mL 5 wt% NH_3_ in ultrapure water, argon flow of 30 mL min^−1^, 30 °C, and 525 kHz) [50]. Note that a 5 wt% NH_3_ solution in ultrapure water was previously found to be a good compromise between the formation rate of hydrazine and cavitation dynamics and was therefore selected here as the medium composition. Under these conditions, calorimetry experiments indicated an acoustic power density of 0.36 W mL^−1^ (i.e., two times higher than in our previous investigation). Since NH_3_ degasses from the solution during ultrasonic irradiation, and also because this degassing is not linear over time, it was difficult to accurately determine the NH_3_ conversion. For this reason, we focused the discussion on the initial rate of formation of hydrazine, which serves as a reference result throughout this study. Hydrazine yields were measured by spectrophotometry (ESI, Figure S2). The hydrazine formation rates reported throughout this article are given with an uncertainty of ±10%. Under these conditions, hydrazine was produced at a rate of 0.59 mmol L^−1^ h^−1^. This value is 3.7 times higher than the rate previously reported [49, 50], which can be attributed to the higher acoustic power density delivered by the current reactor (0.36 W mL^−1^ vs. 0.17 W mL^−1^ in our earlier work). To verify the potential role of trace amounts of N_2_ or air in hydrazine formation, control experiments were conducted by bubbling N_2_ through ultrapure water (i.e., without NH_3_). In these experiments, hydrazine was no longer detected, confirming that hydrazine is derived from NH_3_.

**FIGURE 1:**
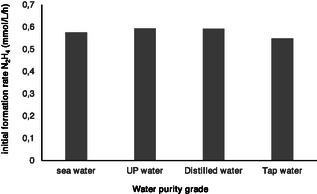
Effect of the water purity grade on the initial hydrazine formation rate. Reaction conditions: 5 wt% aqueous NH_3_ (100 mL), 525 kHz, 45 °C, and 30 mL/min of Ar.

With this reference reaction in our hands, we next evaluated the influence of water purity on the initial rate of hydrazine formation (Figure 1). According to the Rayleigh*–*Plesset equation, impurities in water can affect the dynamics of cavitation bubbles by altering, for example, the liquid surface tension or the solubility of gases in the liquid phase [48, 51, 53]. In this context, the sonochemical conversion of NH_3_ was examined in distilled water, tap water (from Poitiers, France), and even seawater (collected in Royan, France). The results were then compared with those obtained in ultrapure water. To our satisfaction, water purity did not significantly impact the hydrazine formation rate, which remained in the range of 0.55–0.58 mmol L^−1^ h^−1^ (Figure 1). This finding is noteworthy, as seawater is far more abundant and cheaper than ultrapure water that is usually recommended for sonochemical reactions. Additional information regarding the role of salts present in water is provided below.

**FIGURE 2:**
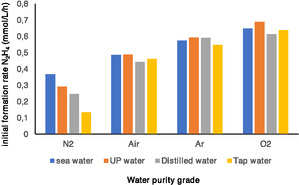
Impact of the gas atmosphere and water purity grade on the initial formation rate of hydrazine (100 mL 5 wt% aqueous NH_3_, 525 kHz, 45 °C, and gas flow: 30 mL/min).

A similar conclusion was reached when the gas atmosphere was varied (Figure 2). Under O_2_, Ar, and air, the quality of the water (ultrapure, distilled, tap, or seawater) had no significant influence on the initial hydrazine formation rate. Only in the case of nitrogen, seawater yields the highest hydrazine formation rate. At this stage, we have no explanation to rationalize this particular behavior observed under N_2_. On the other hand, regardless of water purity, the nature of the gas atmosphere does influence the initial rate of the formation of hydrazine. The highest rate was observed under oxygen (0.69 mmol L^−1^ h^−1^), followed by argon (0.59 mmol L^−1^ h^−1^), air (0.49 mmol L^−1^ h^−1^), and nitrogen (0.49 mmol L^−1^ h^−1^) (Figure 2). The impact of the gas atmosphere on hydrazine formation can be attributed to (i) differences in gas solubility in aqueous NH_3_ solutions, (ii) the intrinsic physicochemical properties of the gases (thermal conductivity, heat capacity, etc.), and (iii) differences in the amount of in situ oxygenated radicals formed, all of which are known to affect cavitation bubble dynamics and the associated reactivity [48]. More information on these aspects is provided later.

To tentatively rationalize these results, in particular those unexpectedly obtained in seawater, we examined the influence of salts present in water on the initial rate of hydrazine formation. As a first approximation, the presence of salts is expected to alter the surface tension of water [54, 56], a physicochemical parameter capable of affecting cavitation bubble size as well as the local pressure and temperature reached during bubble collapse. Additionally, salts are known to interact with the hydrogen‐bonding network in water, thereby decreasing gas solubility, known as salting‐out effect [57, 59]. In general, the higher the ionic valence and the greater the increase in surface tension, the stronger the salting‐out effect.

**FIGURE 3:**
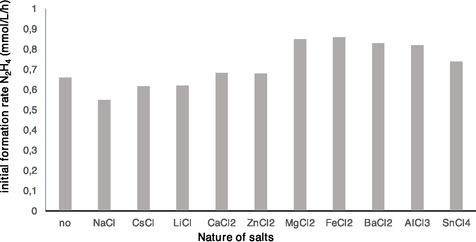
Initial formation rate of hydrazine as a function of salts present in water (100 mL 5 wt% aqueous NH_3_, 525 kHz, 45 °C, 30 mL/min of Ar, 0.04 M of salt, and mechanical stirring at 300 rpm).

In this context, the initial hydrazine formation rate was re‐examined in 5 wt% ultrapure aqueous NH_3_ solutions containing 0.04 M of monovalent (NaCl, LiCl, and CsCl), divalent (MgCl_2_, BaCl_2_, ZnCl_2_, CaCl_2_, and FeCl_2_), or trivalent (AlCl_3_) ions. These experiments were conducted under otherwise identical conditions, in an Ar flow of 30 mL min^−1^. To avoid any mass‐transfer limitations in the presence of salts (e.g., concentration gradients), all aqueous solutions were mechanically stirred at 300 rpm during ultrasonic irradiation. In ultrapure water, the mechanical stirring did not significantly impact the initial rate of hydrazine formation (from 0.58 to 0.60 mmol L^−1^ h^−1^, i.e*.* *,* within the experimental range of error of ±10%) during ultrasonic irradiation. The results are shown in Figure 3.

Compared with ultrapure water, the addition of monovalent ions such as NaCl, LiCl, and CsCl did not significantly affect the initial rate of hydrazine formation, corroborating the feasibility of using seawater, which is rich in monovalent ions (Na^+^ alone accounts for 80% of total ions; Figure S3) (Figure 3). In contrast, a systematic increase in the initial rate of hydrazine formation was observed in the presence of multivalent ions. The highest rates were obtained with MgCl_2_, BaCl_2_, FeCl_2_, and AlCl_3_, for which the hydrazine formation rate increased to as much as 0.85 mmol L^−1^ h^−1^. Because multivalent ions are known to exhibit strong salting‐out effects [57, 59], this rate enhancement of the hydrazine formation cannot be attributed to a greater solubility of Ar in the aqueous NH_3_ solution (i.e., more cavitation). On the other hand, measurements using a drop‐shape analyzer (sessile‐drop tensiometer) revealed no significant effect of salts on the surface tension of 5 wt% aqueous NH_3_, at least at the concentration investigated (0.04 M). Overall, these findings indicate that, within our concentration range, the increased hydrazine formation rate observed with multivalent ions cannot be explained by changes in surface tension or argon solubility. Of course, it should be noted that ionic effects may become more significant at higher salt concentrations.

Unlike monovalent ions, we noticed that the dissolution of multivalent ions in a 5 wt% aqueous NH_3_ solution resulted in turbidity and the formation of a white precipitate. This precipitate can be attributed to the formation of insoluble M(OH)_*x*_(Cl)_γ_ species (M = Mg, Ba, Fe, Al) under basic conditions. X‐ray diffraction (XRD) analysis of the white solid recovered after adding MgCl_2_ to the NH_3_ solution confirmed the formation of Mg(OH)_2_ (Figure S4). To evaluate the impact of this precipitate on cavitation, additional experiments were carried out. First, the Mg(OH)_2_ precipitate formed after dissolving MgCl_2_ in the 5 wt% NH_3_ solution was removed by filtration before ultrasonic irradiation. This removal led to a significant 2.2‐fold decrease in the initial hydrazine formation rate (Table 1, entries 1–3), suggesting that the in situ formed Mg(OH)_2_ particles influence cavitation. In line with this interpretation, when the isolated Mg(OH)_2_ precipitate was resuspended in an ultrapure 5 wt% NH_3_ solution (i.e., without MgCl_2_), the rate of hydrazine formation increased markedly and reached values comparable to those observed in the presence of MgCl_2_ (Table 1, entry 4).

**TABLE 1:** Impact of Mg‐based particles in the formation rate of hydrazine.

Entry	Solution composition before ultrasonic irradiation	Formation rate of hydrazine, mmol L^−1^ h^−1^
1	UP	0.58
2	UP + MgCl_2_ a	0.85
3	UP + MgCl_2_ a + filtration	0.38
4	UP + Mg(OH)_2_ b	0.85

a 0.04 M.

b The entire amount of recovered Mg(OH)_2_ in entry 3 was introduced in entry 4.

**FIGURE 4:**
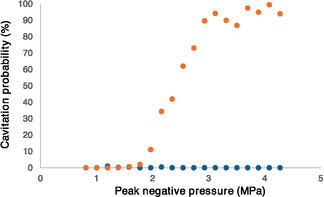
Cavitation probability measurements without (blue circle ●) and with Mg(OH)_2_ particles (orange circle ●) (100 mL 5 wt% aqueous NH_3_, 525 kHz, 45 °C).

Therefore, we hypothesize that the Mg(OH)_2_ particles suspended in the NH_3_ aqueous solution during ultrasonic irradiation may stabilize gas nuclei, thus facilitating the formation of a larger number of cavitation bubbles. This interpretation is inspired by previous studies showing that the energy required to overcome the liquid surface tension at a solid–liquid interface is significantly lower, thereby resulting in an increased density of cavitation bubbles [60, 62]. To support this hypothesis, cavitation probability measurements were performed in the presence and absence of Mg(OH)_2_ (Figure 4). In both cases, the cavitation threshold, defined as the peak negative pressure at which cavitation probability reaches 50%, was determined. The solution containing Mg(OH)_2_ exhibited a cavitation threshold of 2.5 MPa peak negative pressure, a value substantially lower than that required in ultrapure water (>140 MPa) [63, 64]. Taken together, these findings suggest that Mg(OH)_2_ particles serve as heterogeneous nuclei that promote the formation and growth of cavitation bubbles, thereby enhancing the hydrazine formation rate. It should be noted that no changes in the XRD patterns of Mg(OH)_2_ were observed after ultrasonic irradiation. However, we cannot rule out the possibility that, even though physical effects are limited at high ultrasonic frequencies, re‐dissolution, precipitation, or agglomeration of Mg(OH)_2_ particles may occur over time during ultrasonic irradiation, a phenomenon that could also alter cavitation dynamics [65]. Nevertheless, this does not affect our conclusion that these precipitated Mg(OH)_2_ particles initially reduce the cavitation threshold. The slight differences in performance between the Mg, Ba, Al, Fe group and the Ca, Zn group may arise, in a first approximation, from differences in their speciation in aqueous NH_3_, leading to varying degrees of precipitate formation, which was also observed visually (Figure S5). It should be noted that other aspects (not explored here) such as particle size, surface composition (e.g., trapped gas pockets, crevices, etc.), and surface polarity may also influence the nucleation of cavitation bubbles on particles [66].

### 2.2 Nitrite and Nitrate Formation

**FIGURE 5:**
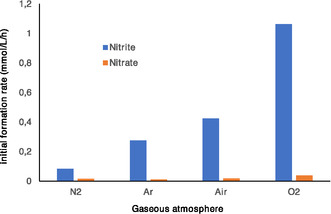
Initial formation rate of ammonium nitrate and nitrite as a function of the gaseous atmosphere (100 mL 5 wt% aqueous NH_3_, 525 kHz, 45 °C, and gas flow: 30 mL/min).

In addition to hydrazine, we investigated the formation of ammonium nitrite and ammonium nitrate, which could potentially arise from NH_3_ oxidation and combustion, respectively (Figure 5). Both compounds were analyzed and quantified using ion chromatography, chosen for its high sensitivity. The uncertainty in concentration measurement was ±10% (Figures S6 and S7). To prevent ionic interference during analysis, all reactions were carried out in ultrapure water.

Ammonium nitrate is typically produced *via* the Ostwald process [67], which involves burning NH_3_ at 900 °C and under pressure of O_2_ (∼10 bar). These conditions could theoretically be reached during cavitation bubble collapse [48]. In our system, O_2_ may be present in the ultrasonic reactor either from the gaseous atmosphere when O_2_ or air is bubbled or, under other atmospheres such as Ar or N_2_, through the disproportionation of HO^●^ radicals (into O_2_ and water) generated by water sonolysis [68]. Although the formation rate of ammonium nitrate was highest under an oxygen atmosphere, it remained approximately 40 times lower than that of hydrazine, suggesting that NH_3_ combustion under ultrasonic conditions is not a dominant pathway (Figure 5).

**FIGURE 6:**
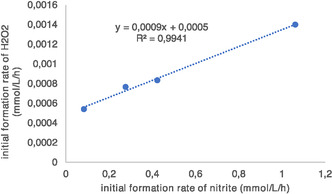
Correlation between the initial formation rate of H_2_O_2_ and that of ammonium nitrite (100 mL 5 wt% aqueous NH_3_, 525 kHz, 45 °C, and 30 mL/min of Ar).

Conversely, ammonium nitrite was detected in significantly higher amounts (Figure 5). The gaseous atmosphere strongly influenced its initial formation rate, which increased from 0.083 to 0.276, 0.423, and 1.062 mmol·L^−1^·h^−1^ as the atmosphere changed from N_2_ to Ar, air, and O_2_, respectively. Ammonium nitrite is typically formed through the oxidation of NH_3_ by in situ generated HO^●^ radicals. To support this proposed reaction pathway, the amount of H_2_O_2_, produced via HO^●^ recombination, was quantified by spectrophotometry in pure water subjected to similar ultrasonic irradiation (Figures S8 and S9). Consistently, the initial formation rate of H_2_O_2_ followed the same trend as ammonium nitrite: 0.00054 mmol·L^−1^·h^−1^ under N_2_  <  0.0076 mmol·L^−1^·h^−1^ under Ar  <  0.0083 mmol·L^−1^·h^−1^ under air  <  0.014 mmol·L^−1^·h^−1^ under O_2_. A decent linear correlation was even observed between the initial formation rates of H_2_O_2_ and ammonium nitrite, providing evidence that ammonium nitrite is most likely formed *via* NH_3_ oxidation by HO^●^ radicals during cavitation bubble collapse (Figure 6).

Given this oxidative pathway, it is quite surprising that ammonium nitrite was formed as a major oxidized product, as it is expected to be further oxidized to ammonium nitrate in the presence of HO^●^ radicals. This result can be explained by the difference of interactions of in situ generated nitrogen species at the cavitation bubble‐water interface. Our previous work has shown that the interaction of a solute at the cavitation bubble interface is mainly driven by hydrophobic interactions, with logP serving as a reliable descriptor [69]. As a salt, ammonium nitrite is highly hydrophilic and therefore does not strongly interact with the cavitation bubbles. Instead, it remains in the bulk water solution, where it may potentially react with HO^●^ radicals diffusing from the cavitation bubbles into the bulk solution. In contrast, NH_3_, being a gas, fills the cavitation bubbles. We hypothesize that most HO^●^ radicals are scavenged by NH_3_ (to form H_2_N^●^, Δ*G*= −46 kJ mol^−1^) [70] at the cavitation bubble interface, leading to the formation of ammonium nitrite (initiated by the formation of H_2_N^●^ with HO^●^) as a primary product. As a hydrophilic salt, ammonium nitrite rapidly diffuses into the bulk water solution, where it is protected from oxidation due to the rapid scavenging of HO^●^ radicals by NH_3_ at the cavitation bubble interface. Note that a same reasoning applies to explain the accumulation of hydrazine in the reactor. A rather similar effect has been previously observed in electrochemistry, where high concentrations of NH_3_ limit overoxidation of ammonium nitrite to ammonium nitrate [71]. To validate this hypothesis, a sodium nitrite solution was subjected to ultrasonic irradiation in the absence of NH_3_. As expected, and in contrast to observations in the presence of NH_3_, nitrites were converted to nitrates over time, confirming that NH_3_ does protect ammonium nitrite from further oxidation. This result is also in line with one of our previous results showing that sonochemical conversion of NH_3_ suppressed the formation of H_2_O_2_ typically formed during ultrasonic irradiation of pure water [50]. Further evidence supporting the predominant formation of ammonium nitrite is provided later in the section dedicated to gas phase analysis.

**FIGURE 7:**
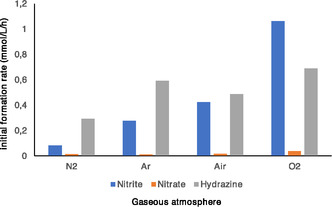
Initial formation rate of hydrazine, ammonium nitrate, and ammonium nitrite as a function of the gaseous atmosphere (100 mL 5 wt% aqueous NH_3_, 525 kHz, 45 °C, and 30 mL/min of Ar).

Interestingly, by adjusting the gaseous atmosphere, the sonochemical conversion of NH_3_ can be directed predominantly toward either hydrazine or ammonium nitrite (Figure 7). For example, under O_2_ or air, the initial rate of formation of ammonium nitrite exceeds that of hydrazine (1.5 times higher in O_2_), which can be explained by the increased generation of HO^●^ and HOO^●^ radicals (Figure S9) [72]. In contrast, under oxygen‐poor atmospheres such as N_2_ or Ar, the initial formation rate of hydrazine becomes higher than that of ammonium nitrite (3.5 times higher under N_2_), thus providing a means to tune the reaction selectivity.

With these results in our hands, hydrogen gas was bubbled into the solution to tentatively reduce the oxidative reaction that forms ammonium nitrite (Table 2). H_2_ is known to dissociate inside cavitation bubbles [72], generating H^●^ radicals that react with HO^●^ radicals at the bubble interface to produce water, a competing pathway that should partially inhibit the HO^●^‐mediated oxidation of NH_3_ to ammonium nitrite [72]. However, when pure hydrogen was bubbled at a flow rate of 30 mL/min, the formation of both hydrazine and ammonium nitrite completely seized (Table 2, entry 2). This observation can be attributed to the very low solubility of H_2_ in water, which prevents efficient cavitation, and/or to its high heat capacity leading to a decrease of the temperature released at the cavitation bubble implosion. To overcome this limitation, mixtures of Ar and H_2_ were bubbled through a 5 wt% aqueous NH_3_ solution. Gratifyingly, with a 50/50 Ar/H_2_ mixture, the initial hydrazine formation rate decreased only slightly, from 0.59 to 0.50 mmol·L^−1^·h^−1^, while the initial formation rate of ammonium nitrite dropped dramatically from 0.28 to only 0.06 mmol·L^−1^·h^−1^ (Table 2, entry 3). When the H_2_ content in Ar was further increased, cavitation was however dramatically reduced (Table 2, entry 4). Overall, these results demonstrate that reaction selectivity can be tuned by adjusting the gas atmosphere.

**TABLE 2:** Impact of H_2_ on the initial formation rate of hydrazine and ammonium nitrite.^a^

Entry	Gas atmosphere	Hydrazine formation rate, mmol L^−1^ h^−1^	Ammonium nitrite formation rate, mmol L^−1^ h^−1^
1	Ar	0.59	0.28
2	H_2_	—	—
3	Ar/H_2_ (50/50)	0.50	0.06
4	Ar/H_2_ (20/80)	<0.1	—

a Reaction conditions: 5 wt% aqueous NH_3_ (100 mL), 525 kHz, 45 °C, and 30 mL/min of gas.

### 2.3 Gas Phase

Finally, the gas phase products were analyzed by directly connecting the gas outlet to a gas chromatography (ESI). For all experiments, argon was continuously bubbled at a flow rate of 30 mL·min^−1^. The formation of H_2_ was detected, with an initial rate of formation of 0.14 mmol·h^−1^, approximately five times and two times lower than the rates of formation of hydrazine and nitrite, respectively, indicating that the decomposition of NH_3_ to N_2_ and H_2_ is not a dominant pathway. Once again, and as discussed above for ammonium nitrite, this result supports the compartmentation of hydrazine into the bulk water solution during sonochemical conversion of NH_3_. Since H_2_ can also originate from water sonolysis, a control experiment using ultrapure water was performed to assess the contribution of water sonolysis to the overall H_2_ production. From these control experiments, we can conclude that, in addition to water sonolysis, additional hydrogen is generated from NH_3_ activation. However, distinguishing between hydrogen originating from water sonolysis and that from NH_3_ decomposition remains challenging at this stage. Indeed, reactions induced by cavitation bubbles involve competitive processes, self‐annihilation, and feedback mechanisms that may be more complex than a simple linear addition.

In addition to H_2_ and N_2_, NO_*x*_ species are also expected in the Ar gaseous phase. However, our online gas chromatography, which is connected to the ultrasonic reactor, is unable to detect these compounds. Therefore, the gas phase product was further analyzed online using fourrier transformation‐Infrared (FT‐IR) spectroscopy, a well‐established method for identifying NO_*x*_ formation [73]. While all FT‐IR spectra obviously exhibit a strong ammonia signal, the formation of N_2_O was unambiguously identified at 2224 cm^−1^, corresponding to its ν_3_ asymmetric stretching mode (Figure S10). This observation is fully consistent with the formation of ammonium nitrite in the liquid phase. Indeed, ammonium nitrite is known to decompose to N_2_O. Other NO_*x*_ such as NO, NO_2_, or N_2_O_4_, which are typical makers of the presence of nitrate, were not detected by FT‐IR, further confirming the selective sonochemical conversion of NH_3_ to ammonium nitrite with HO^●^ radicals. It should be noted that, unlike under an Ar atmosphere, N_2_O was no longer detected under an Ar/H_2_ (50/50) atmosphere, further confirming the selective sonochemical conversion of ammonia to hydrazine under this gaseous atmosphere (Figure S11).

### 2.4 Estimation of the Energy Consumption

Finally, to roughly assess the energy consumption of this technology, we conducted calorimetry experiments under argon to estimate the acoustic power density delivered to the solution [74], which was approximately 0.36 W/mL (i.e., 36 W in 100 mL of 5 wt% aqueous NH_3_ solution). Considering the initial formation rates of hydrazine and ammonium nitrite (as shown in Figure 7), and consistent with our previous articles [50, 75], this corresponds to an energy efficiency (defined as the ratio of the N─H bond dissociation energy of NH_3_ to the delivered acoustic energy) of less than 1%. This value is comparable, for example, to the energy efficiency of H_2_ production *via* photocatalytic water splitting [76, 77]. This low energy efficiency underscores the current challenges faced by these alternative technologies in terms of scalability, in particular for the conversion of refractory small molecules such as NH_3_ [78].

Regarding scale‐up, it is important to note that our ultrasonic reactor was not designed for this purpose; it was designed and used to understand the reaction mechanism. In this type of ultrasonic reactor, the formation of cavitation bubbles is very often the rate‐limiting step, with the cavitation bubble cloud remaining in the proximity of the transducer, and most of the water volume is used solely to dissipate heat [75, 79]. In our previous reports, we demonstrated that reducing the ratio of the sonicated solution volume to the transducer surface area resulted in an exponential increase in reaction rates and reactor productivity, bringing energy consumption much closer to industrial standards in the field [69, 78]. In other words, transitioning from batch to microfluidic reactors offers promising opportunities for improvements in energy efficiency and scale‐up.

## 3 Conclusion

Here, we report the main nitrogen‐based products formed through the sonochemical activation and conversion of NH_3_. Analysis of the liquid phase revealed the dominant formation of hydrazine, which mainly results from the cleavage of the N─H bond of NH_3_, and ammonium nitrite formed by oxidation of NH_3_ with in situ produced HO^●^ radicals stemming from the concurrent sonolysis of water. Partial decomposition of NH_3_ to H_2_ was also observed but remains a minor side reaction, thanks to the compartmentation of hydrazine and ammonium nitrite into the bulk water solution during sonochemical conversion of NH_3_. Advantageously, by modifying the gas atmosphere, we discovered in this work a means to control the selectivity of the reaction toward either hydrazine (e.g., under Ar/H_2_ atmosphere) or ammonium nitrite (under oxygen atmosphere).

Furthermore, in 5 wt% aqueous NH_3_ solution, we revealed that the presence of multivalent salts, such as MgCl_2_, enhances the initial rate of formation of hydrazine. This effect was rationalized by the in situ precipitation of Mg(OH)_2_ particles, which act as cavitation agent. These particles promote cavitation by drastically reducing the threshold for the nucleation of cavitation bubbles (2.5 MPa negative pressure).

From a fundamental standpoint, this work demonstrates that it is possible to harness the energy released at the cavitation bubbles collapse time to activate and selectively convert NH_3_, without the assistance by any catalyst or preformed cavitation agent. Ongoing research within our group focuses on the development of microfluidic ultrasonic reactors to assess and reduce the energy consumption of this technology and enable its scale‐up.

## Funding

This work was supported by the Centre National de la Recherche Scientifique, Université de Poitiers, France2030 (ANR‐22‐PESP‐0006).

## Conflicts of Interest

The authors declare no conflicts of interest.

## Supporting information

Supplementary Material

## Data Availability

The data that support the findings of this study are available from the corresponding author upon reasonable request.
