# Supplementary material for: Tunable Conversion of Ammonia to Hydrazine or Ammonium Nitrite Induced by Acoustic Cavitation Bubbles
Source: ChemSusChem. 2026 Jul 5;19(13):e70847. doi: 10.1002/cssc.70847 (PMC13333598; doi:10.1002/cssc.70847)
Supplement: Supplementary file 1 — Supplementary Material [file CSSC-19-e70847-s001.pdf]

SUPPORTING INFORMATION

Tunable conversion of ammonia to hydrazine or ammonium nitrite induced by acoustic cavitation bubbles

Damien Denis,[a] Zhangyue Xie,[b] E. Fourré,[a] P. N. Amaniampong,[a] Wen Liu,[b] and François Jerome*[a]

Description of the ultrasonic reactor

Experiments were performed on a 525 kHz ultrasonic reactor purchased from Sinaptec Ultrasonic Technology (ultrasonic generator NexTgen HF GUI, $P_{electric}$= 100 W). This reactor is equipped with three piezoelectric materials which generate ultrasonic waves at a frequency of 525 kHz. During ultrasonic irradiation, piezoelectric materials were continuously cooled down thanks to the flow of air. The temperature of the solution was monitored with a thermocouple and maintained at 47°C thanks to a cooling jacket.

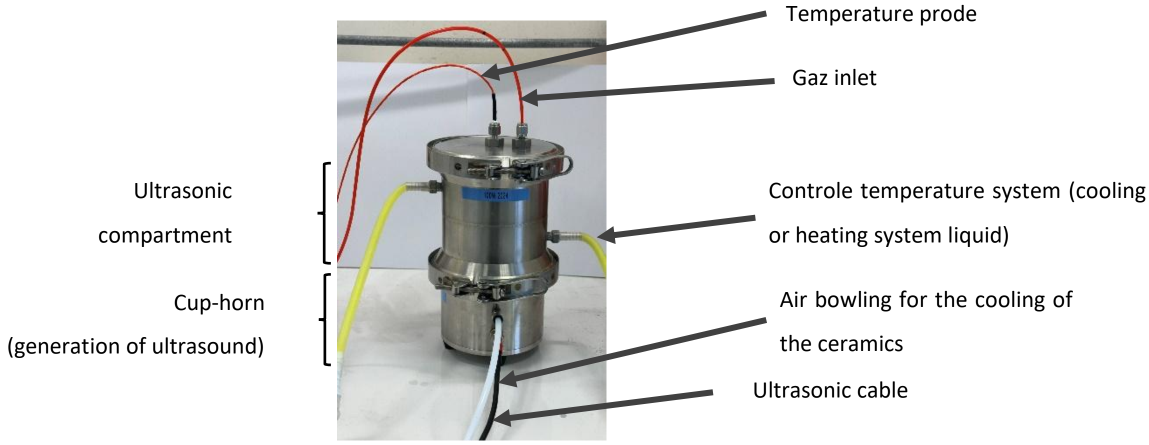

Figure S1. Picture the ultrasonic reactor employed thorough this study

Typical procedure for the sonolysis of 5 wt% aqueous NH_3

In a standard procedure, 100 mL of a 5 wt% aqueous NH_3 solution was introduced into the ultrasonic reactor. Prior to initiating ultrasonic irradiation, Ar was bubbled through the solution at a flow rate of 30 mL·min^{-1} for 20 minutes to remove dissolved air. Ultrasonic irradiation was then applied at a frequency of 525 kHz and a power density of 0.36 W·mL^{-1}. Throughout the irradiation process, Ar bubbling was maintained at the same flow rate (30 mL·min^{-1}), and the solution temperature was kept constant at 45 °C. by continuously circulating a cooling liquid (at 30 °C) through the reactor jacket a Huber 300H ole minichiller. During the reaction, aliquots were periodically sampled and analyzed *via* spectrophotometry and ion chromatography to monitor the formation rates of hydrazine, ammonium nitrate, and ammonium nitrite.

Note: This procedure was adapted when atmospheres (N_2, O_2, air) were varied and/or when salts were added.

Quantification of hydrazine

Hydrazine was quantified using a UV visible spectrophotometer (ThermoFisher Evolution 60S). The principle of this titration is based on the derivatization of hydrazine with *p*-dimethylaminobenzaldehyde (DMAB) under acid conditions, yielding a yellow hydrazone compound which adsorb at 458 nm. The following procedure has been typically used:

- *Preparation of the DMAB solution (0,2 M in HNO_3 1 M):* 14.9 g of DMAB were dissolved in 30 mL of 65% aqueous HNO_3. The volume of the solution was then adjusted to 500 mL by addition of ultrapure water.
- *Preparation of the standard solutions of hydrazine:* hydrazine standard solutions were prepared by dilution of a mother solution of 100 mg/L of hydrazine monohydrate in water. The concentration range of the hydrazine standards were in a 0-21 mg/L window.
- *Protocol for titration:* 100 µL of the hydrazine solution (0.1-20 mg/L) were mixed with 2.5 mL of the 0.2 M DMAB solution in HNO_3 (1 M). The absorbance was measured at 458 nm on a UV-visible spectrophotometer (ThermoFisher Evolution 60S). The calibration curve (absorbance as a function of the hydrazine concentration) is presented in Figure S2 and was used to quantify the amount of hydrazine formed during ultrasonic irradiation of NH_3.

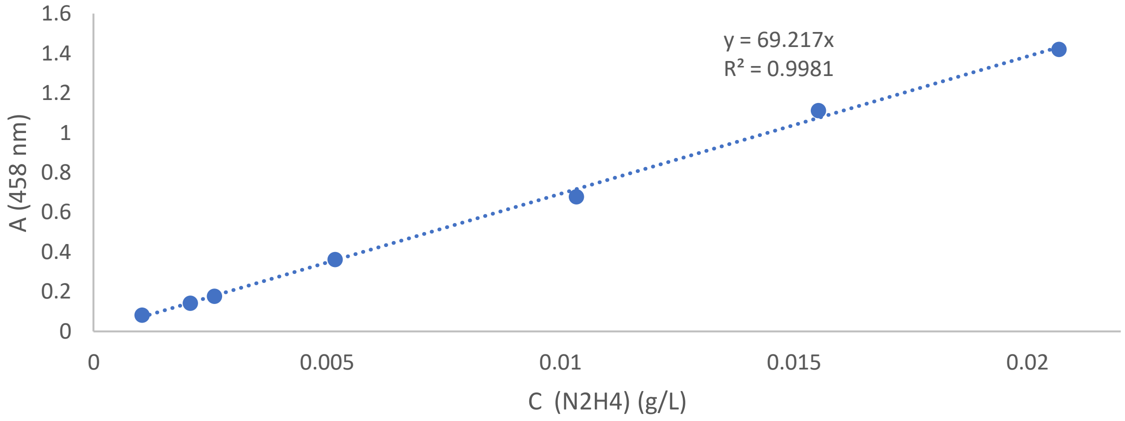

Figure S2. Plot of the absorbance as a function of the hydrazine concentration

	λ *(en nm)*	mg/L	%RSD		λ *(en nm)*	mg/L	%RSD		λ *(en nm)*	mg/L	%RSD
Ag	328,068	< 0,05		Ga	294,363	< 0,05		Zn	213,857	< 0,05	
Al	396,152	< 0,05		K	766,491	316	*0,59*	B	249,772	2,30	*1,58*
As	188,980	< 0,05		Li	670,783	0,29	*0,81*	Ge	209,426	< 0,05	
Ba	455,403	< 0,05		Mg	279,553	844	*1,89*	Mo	202,032	< 0,05	
Be	313,042	< 0,05		Mn	257,61	< 0,05		Nb	313,078	< 0,05	
Bi	223,061	< 0,05		Na	589,592	5591	*0,48*	P	213,618	< 0,05	
Ca	396,847	291	*0,75*	Ni	231,604	< 0,05		S	181,972	440	*0,55*
Cd	214,439	< 0,05		Pb	220,353	< 0,05		Si	251,611	3,36	*0,28*
Co	238,892	< 0,05		Se	196,026	< 0,05		Ta	268,517	< 0,05	
Cr	267,716	< 0,05		Sr	407,771	5,02	*0,78*	Ti	336,122	< 0,05	
Cu	327,395	< 0,05		Tl	190,794	< 0,05		W	207,912	< 0,05	
Fe	238,204	< 0,05		V	292,401	< 0,05		Zr	343,823	< 0,05	

Figure S3. Composition of sea water (from Royan in France) determined by ICP.

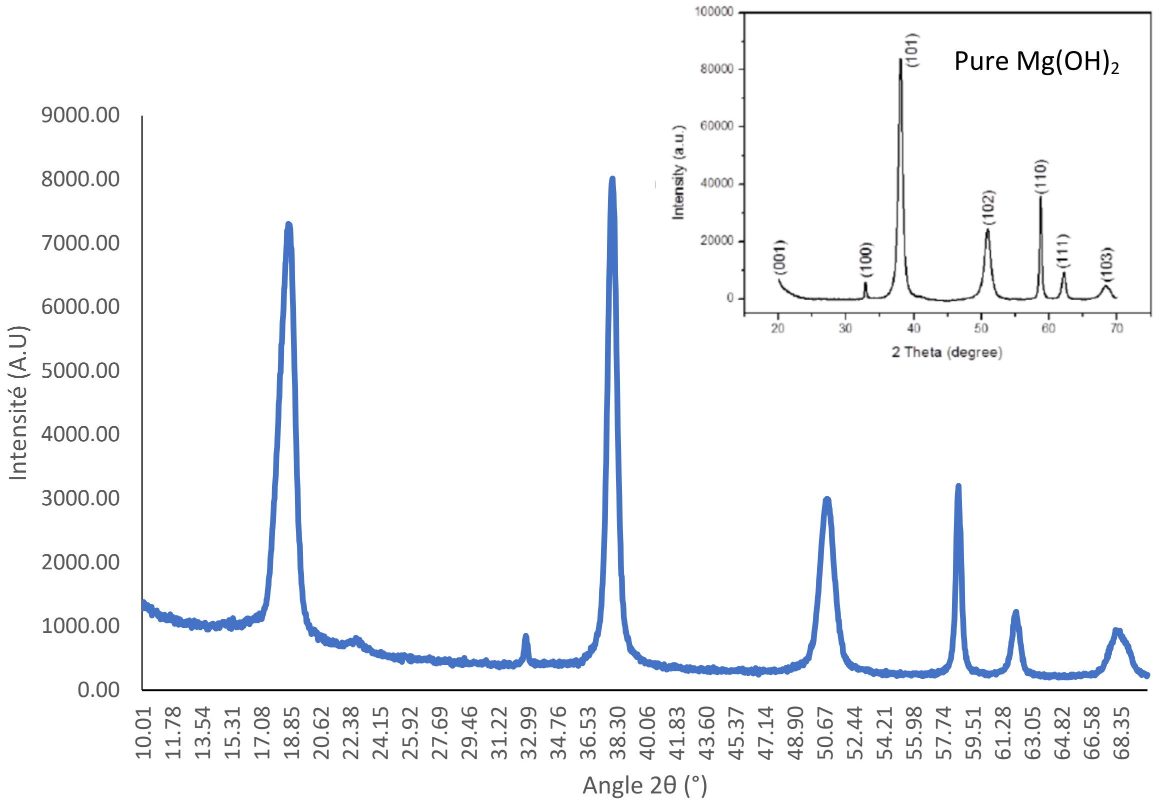

Figure S4. XRD pattern of $Mg(OH)_2$ recovered after $MgCl_2$ was dissolved into 5 wt% aqueous NH_3

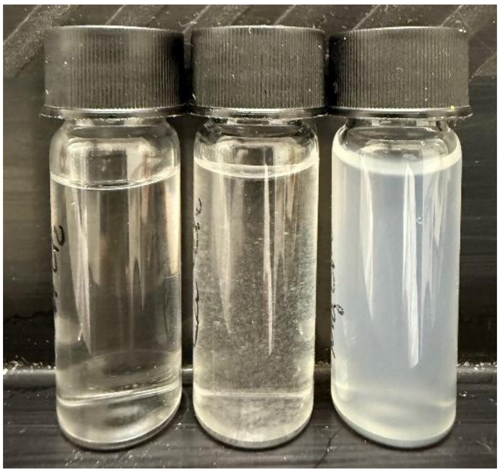

Figure S5. Photos showing the differences in precipitate amounts formed by adding (a) $ZnCl_2$, (b) $CaCl_2$ and (c) $MgCl_2$ into aqueous ammonia (5 wt % in water)

Quantification of ammonium nitrate and nitrite

Ammonium nitrate and nitrite were analyzed on an ion chromatography instrument from Thermo Fisher Scientific (Dionex Aquion system) equipped with a Dionex IonPac AS22 Analytical column (4 x 250 mm) and ion conductivity detector (applied electric current: 41 mA). The eluent (1 L) was prepared by mixing 9 mL of an aqueous solution of sodium carbonate (0.5 M) with 2.8 mL of an aqueous solution of sodium Bicarbonate (0.5 M) and 988.2 mL of ultrapure water. The pump flow rate was set to 1.2 mL/min. The injection volume is 4 µL. The suppressor used is an AERS 4 mm, operated at a current of 41 mA. The column temperature was continuously maintained at 30 °C.

Nitrite standard solutions were prepared from commercial $NaNO_2$, with a final concentration in a 24-300 mg/L range. The calibration curve is shown below.

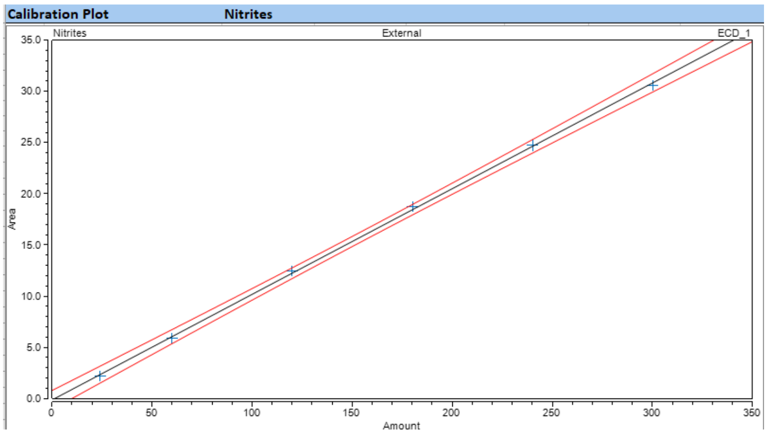

Figure S6. Calibration curve for the quantification of ammonium nitrite by ion chromatography, showing 95% confidence intervals.

Nitrate standard solutions were prepared from commercial $NaNO_3$, with a final concentration in a 24-50 mg/L range. The calibration curve is shown below.

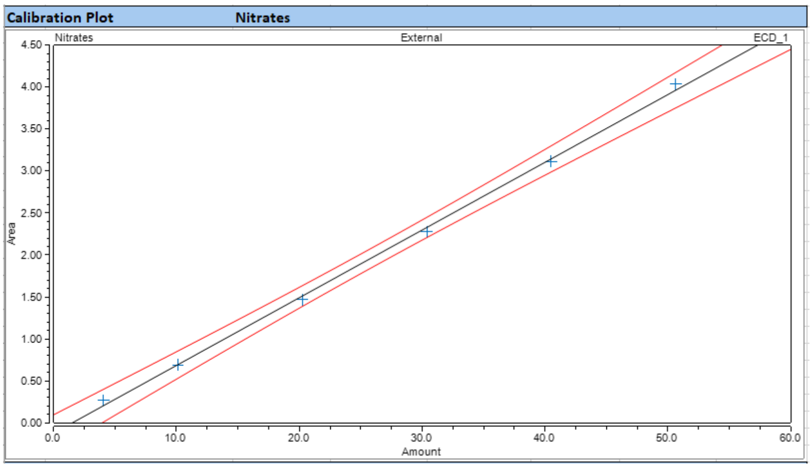

Figure S7. Calibration curve for the quantification of ammonium nitrate by ion chromatography, showing 95% confidence intervals.

Quantification of hydrogen peroxide

Hydrogen peroxide was quantified by spectrophotometry using a UV visible spectrophotometer (ThermoFisher Evolution 60S). The principle of this titration is based on the reaction of hydrogen peroxide with TiO^{2+}, yielding a yellow-orange complex Ti(IV)-H_2O_2 which adsorb at 412 nm. The following procedure has been typically used:

- *Preparation of $TiOSO_4$ solution 0.02 M in H_2SO_4 0.5 M:* 0.552 g of titanium oxysulfate ($TiOSO_4$) was mixed with 2.8 mL of H_2SO_4 (18 M). The solution was then diluted with water to reach a final volume of 100 mL.
- *Preparation of standards solutions of H_2O_2:* hydrogen peroxide standard solutions were prepared from a mother commercial solution of H_2O_2 (30 wt% in water). Standard hydrogen peroxide solutions were prepared in a 0-0.002 mol/L concentration range.
- *Protocol for titration:* 0.5 mL of H_2O_2 solution was mixed with 0.5 mL of the $TiOSO_4$ solution. The absorbance was measured at 412 nm. The calibration curve (absorbance as a function of the hydrogen peroxide concentration) is presented in Figure S7 and was used to quantify the amount of hydrogen peroxide formed during ultrasonic irradiation of NH_3

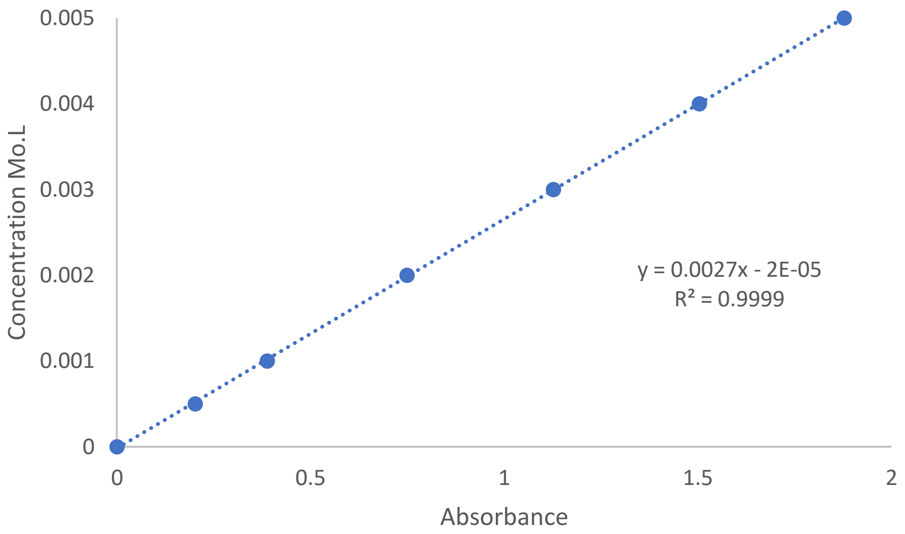

Figure S8. Plot of the hydrogen peroxide concentration as a function of the Ti(IV)-H_2O_2 complex absorbance

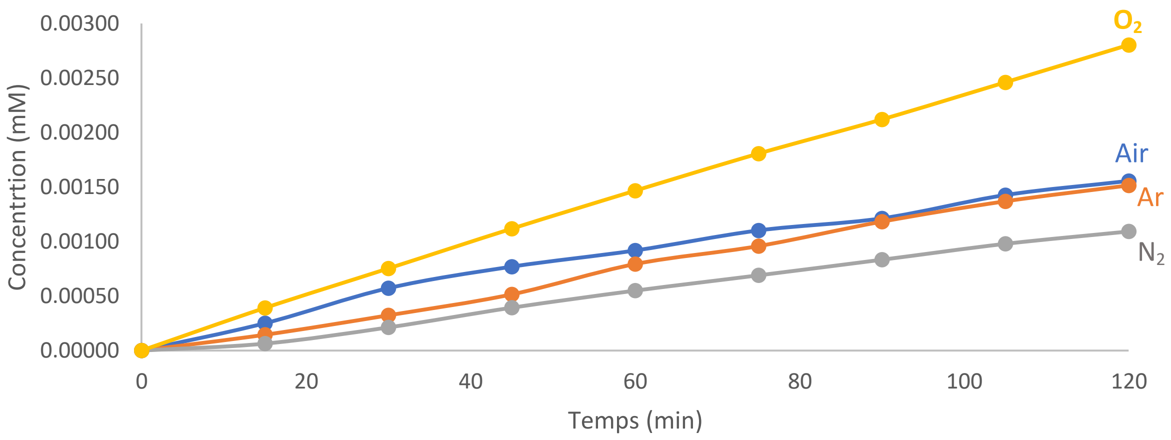

Figure S9. Plot of the hydrogen peroxide concentration as a function of time, and over different gas atmosphere (30 mL.min^{-1}, 30 °C, 525 kHz, 0.36 W.mL^{-1})

Online gas chromatography analysis of H_2 contained in the gas phase

The gaseous products formed during the ultrasonic irradiation of ammoniac were monitored online using a Micro GC Fusion Gas Analyzer (INFICON) connected to the gas outlet of the ultrasonic reactor. Gas sampling was performed directly at the reactor outlet through a heated injection loop (90 °C) to prevent condensation. The GC was equipped with a capillary Rt-Msieve 5A column, (30 m, 0.53 mm ID, 50 µm; column temperature 80 °C; carrier gas: Ar) for H_2 analysis. Detection was performed using a thermal conductivity detector (TCD) at 70 °C.

FT-IR online analysis of NOx in the gas phase

FTIR spectra were recorded using a PerkinElmer Frontier spectrometer equipped with a liquid nitrogen-cooled MCT detector and a 10.6 m optical path length gas cell (Cyclone 10, Specac). The gaseous effluent from the ultrasound reactor was monitored online at room temperature under atmospheric pressure. Prior to ultrasonic irradiation, the reactor was purged with Argon, at a flow rate of 30 mL min^{-1} for 30 min. A background spectrum was then recorded under these conditions (ultrasound off). Ultrasound irradiation was then started, and spectra of the gaseous products were first collected after 15 min of operation. Additional spectra were acquired at 15 min intervals up to 120 mins. Spectra were recorded in the 4000–600 cm^{-1} wavenumber range with a spectral resolution of 4 cm^{-1}. Each spectrum corresponds to the co-addition of 5 scans and is presented in absorbance mode after background subtraction and baseline correction.

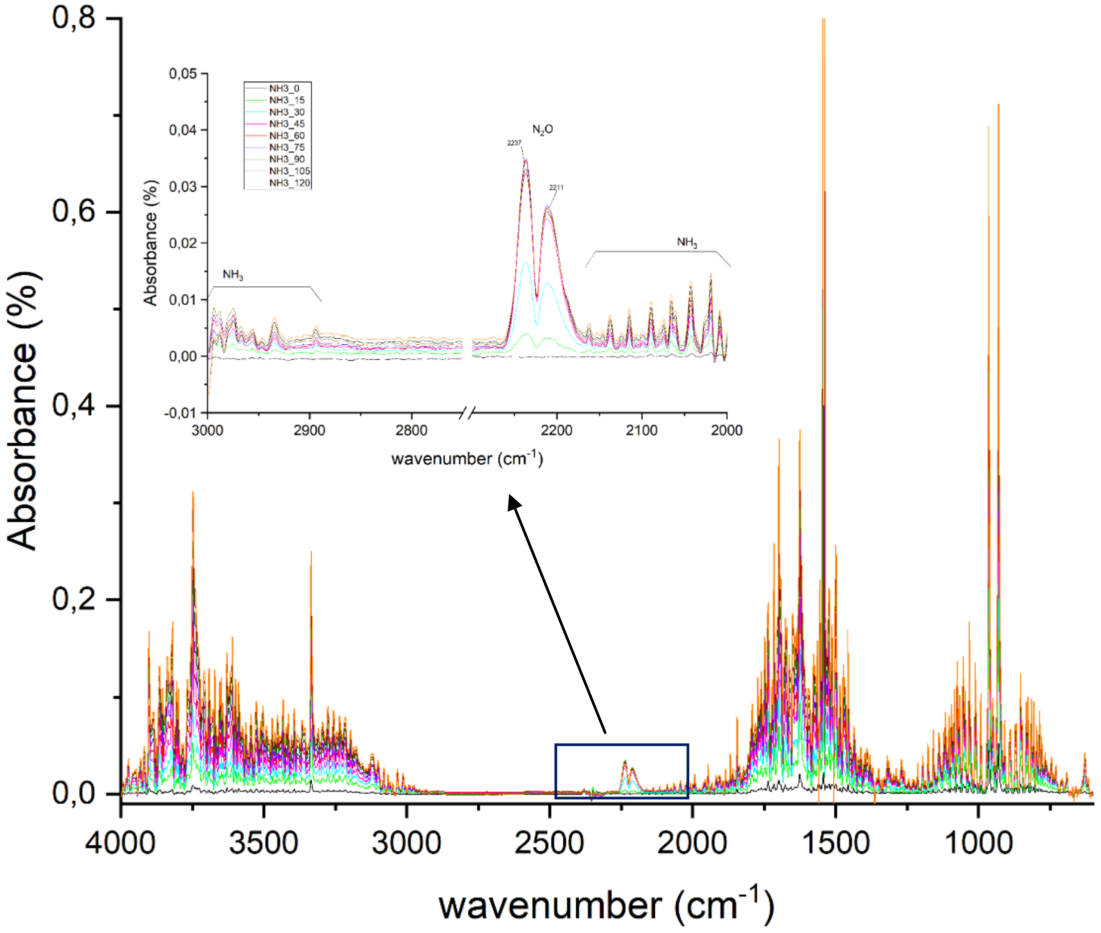

Figure S10: FTIR spectra of gaseous species in the reactor effluent recorded at 15 min intervals after switching on the ultrasound. Spectra were taken every 15 minutes (036 W/mL, 30°C, Ar bubbling at 30 mL/min, 525 kHz) .

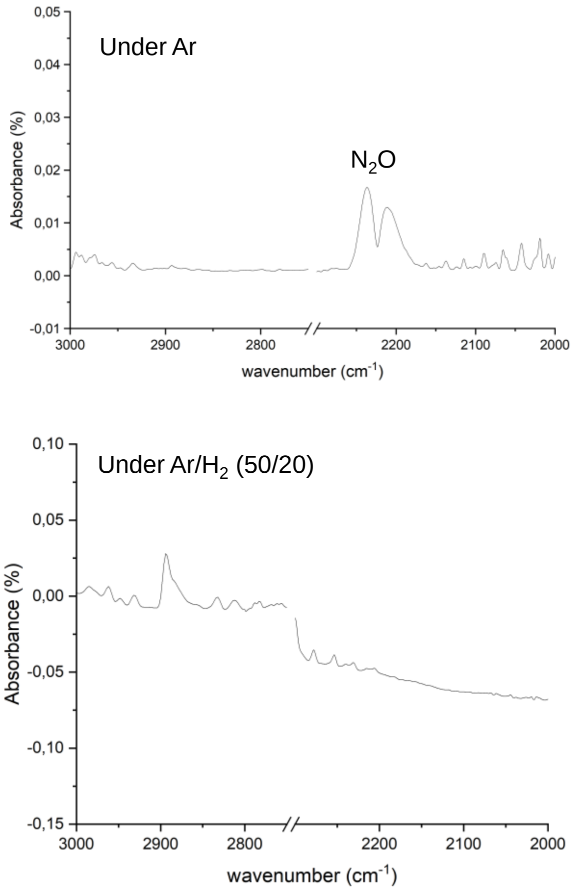

Figure S11: FTIR spectra (zoom on the N_2O region) of gaseous species recorded under (a) Ar and (b) Ar/H_2 (50/50). In (b) the signal was very low suggesting the absence of N_2O in this case. Conditions: 036 W/mL, 30°C, gas bubbling at 30 mL/min, 525 kHz.
